# Sexual phenotype drives variation in endocrine responses to social challenge in a quasi-clonal animal

**DOI:** 10.1098/rsos.180002

**Published:** 2018-04-04

**Authors:** Cheng-Yu Li, Shu-Ping Huang, Mark Garcia, Adam Fuller, Yuying Hsu, Ryan L. Earley

**Affiliations:** 1 Department of Biological Sciences, University of Alabama, 300 Hackberry Lane, Box 870344, Tuscaloosa, AL 35487, USA; 2 Department of Life Science, National Taiwan Normal University, No. 88, Section 4, Tingchou Rd, Taipei 116, Taiwan, Republic of China

**Keywords:** sex differences, hormonal response, social challenge, contest behaviour, *Kryptolebias marmoratus*

## Abstract

In many species, males tend to behave more aggressively than females and female aggression often occurs during particular life stages such as maternal defence of offspring. Though many studies have revealed differences in aggression between the sexes, few studies have compared the sexes in terms of their neuroendocrine responses to contest experience. We investigated sex differences in the endocrine response to social challenge using mangrove rivulus fish, *Kryptolebias marmoratus*. In this species, sex is determined environmentally, allowing us to produce males and hermaphrodites with identical genotypes. We hypothesized that males would show elevated androgen levels (testosterone and 11-ketotestosterone) following social challenge but that hermaphrodite responses might be constrained by having to maintain both testicular and ovarian tissue. To test this hypothesis, we staged fights between males and between hermaphrodites, and then compared contest behaviour and hormone responses between the sexes. Hermaphrodites had significantly higher oestradiol but lower 11-ketotestosterone than males before contests. Males took longer to initiate contests but tended to fight more aggressively and sustain longer fights than hermaphrodites. Males showed a dramatic post-fight increase in 11-ketotestosterone but hermaphrodites did not. Thus, despite being genetically identical, males and hermaphrodites exhibit dramatically different fighting strategies and endocrine responses to contests.

## 1. Background

In many species, the sexes differ in their parental care [1], antipredator behaviour [2], aggression [3,4], and behavioural responses to social challenge [5]. Males tend to behave more aggressively than females, and often become the dominant individual in a group ([6], but see [7]). Females often are aggressive during particular life stages, such as female rodents that show maternal defence against intruders when lactating [8,9]. There is strong evidence that steroid hormones, particularly androgens, oestrogens and glucocorticoids, mediate aggression and contest behaviour across the vertebrates [10–13]. The precise mechanisms that underlie regulation of aggressive behaviour often vary between the sexes [14], and multiple factors such as season (e.g. reproductive status), social context and the timing of hormone production (e.g. acute versus chronic elevations) influence hormone–behaviour relationships in complex and interactive ways [15–17]. For instance, androgen levels predict contest behaviour and social status in both sexes; individuals with higher testosterone levels tend to behave more aggressively and/or achieve higher social status [18,19], but there are exceptions to this ‘rule’ [7]. Oestradiol can promote male-like aggressive behaviour in female rodents when binding to oestrogen receptor α [20,21] and increases the probability of winning contests in females but not males [22]. In male rodents, peripheral inhibition of oestrogen production can increase aggression and enhanced oestrogen production in the central nervous system can inhibit aggression [23]. In birds, however, oestrogen administration can potentiate aggression in males during the non-breeding season [17]. Chronic cortisol treatment inhibits aggression, whereas acute increases in cortisol levels trigger marked increases in aggression [15]. There is thus solid evidence that steroid hormones are closely linked to aggression and dominance status [14], albeit in complicated ways. Many studies also imply sex-specific variation in aggressive behaviour [24,25] and hormone–behaviour relationships. Rarely, however, have the sexes been compared with respect to their neuroendocrine responses to social challenge [5].

Male–male aggressive interactions and hormonal responses to social challenges have been the focus of research in this area, but in many systems females can also be highly aggressive [7–9,26,27]. Although males and females exhibit similar patterns of aggressive behaviour, the sexes might employ different neuroendocrine mechanisms to mediate contest behaviour or in response to social challenge [5,27]. Sex differences often are associated with the expression of genes located on sex chromosomes and their unique effects on physiology and behaviour [28,29]. For example, the sex-determining gene DMY (DM-related PG17 on Y specific) sits on the Y chromosome and is required for the development of many aspects of the male phenotype in medaka (*Oryzias latipes*) [30]. However, genotypic differences (e.g. heterotypic chromosomes and their associated genes) *per se* are not necessary to drive dramatic sex differences, as evidenced by pronounced phenotypic differences between males and females in animals with environmental sex determination [31,32] and in animals that undergo sex change during adulthood [33,34].

We therefore investigated, in an organism with environmental sex determination, whether sex differences in the endocrine response to social challenge would exist despite the sexes possessing the same exact genotype at the same exact time. Such differences would imply that a host of neuroendocrine changes, driven by mechanisms that alter patterns of gene expression, accompany transitions between the sexes. We used mangrove rivulus (*Kryptolebias marmoratus*), one of two vertebrates that reproduce by self-fertilization [35]. Hermaphroditic mangrove rivulus have both functional ovarian and testicular tissue (ovotestis), while males have functional testes only [36]. Primary males result from exposure of embryos to low temperatures; secondary males result from adult hermaphrodites undergoing sex change, which can occur spontaneously or can be induced by high temperatures and short-day photoperiod and involves regression of ovarian and proliferation of testicular tissue [37,38]. This fish's unique reproductive system, in which offspring can be genetically identical to the parent and all siblings, affords the opportunity to investigate endocrine responses to contest experiences in the absence of genetic variation. Mangrove rivulus is an ideal organism for examining behavioural and endocrine responses to social challenge because it is aggressive both in the laboratory and in the field [39] and its contest behaviour is highly correlated with pre-fight hormone levels [40,41].

In males of many species, circulating androgen levels and androgen receptor gene expression in the brain are significantly affected by contest experiences [16,42–44], but relatively little is known about whether females or hermaphrodites would have different neuroendocrine responses to social challenge. In mangrove rivulus hermaphrodites, winning and losing experiences change behaviour without affecting steroid hormone levels [41], and contest experiences alter brain androgen receptor expression only in individuals with low baseline testosterone [45]. It is possible that hermaphroditic mangrove rivulus must precisely regulate hormone status to prevent sex change, which may decrease their fitness; self-fertilization is the predominant mode of reproduction in this species such that, even when given the opportunity to cross with a male, hermaphrodites do so only 6% of the time [46]. Because of this potential constraint, we hypothesized that males and hermaphrodites would exhibit different hormone responses to social conflict. We predicted that males would show increased androgen levels in response to social challenge as a mechanism that primes the individual for future conflict, as has been shown in Mozambique tilapia (*Oreochromis mossambicus)* [42] and California mice (*Peromyscus californicus*) [43]; such a response is not likely to compromise reproductive function or sexual phenotype in males. However, the androgen responses of hermaphrodites to social conflict might be limited because elevated androgens could interfere with their ability to maintain an ovotestis by promoting regression of ovarian tissue (e.g. in Atlantic croaker (*Micropogonias undulatus*), [47]). Rivulus hermaphrodites are significantly more aggressive than males towards model conspecifics [34]; however, whether hermaphrodites and males behave differently during, and exhibit different hormonal responses after, dyadic contests remains unclear. To test this hypothesis, we first measured pre-contest (baseline) steroid hormones (testosterone (T), cortisol (F), oestradiol (E2) and 11-ketotestosterone (11-KT)). We then examined sex differences in contest performance during sex-matched contests involving individuals with identical genotypes. Lastly, we measured post-contest steroid hormones and examined sex differences in the endocrine responses to social challenge.

## 2. Material and methods

### 2.1. Study organism

Mangrove rivulus inhabit mangrove ecosystems from the Caribbean and Yucatan to Florida and the Bahamas [48]. This study used individuals of two isogenic lineages from different geographical areas (DAN2K: Dangriga, Belize, collected in 2000; RHL: San Salvador, Bahamas, collected in 1997), all of which were descendants of individuals collected in the field by D. Scott Taylor. Fish were isolated on the day of hatching and kept individually in 13 × 13 × 10 cm^3^ translucent plastic containers (maintenance container). Two distinct lineages were used to ensure that the findings reported were not restricted to a single lineage. Every container was filled with 750 ml of 25 ppt synthetic seawater (Instant Ocean™) and labelled with a unique code for individual identification. Fish were maintained at 25 ± 2°C on a 12 : 12 photoperiod and fed 2 ml newly hatched brine shrimp (*Artemia*) nauplii at 1500 h every day except between Days 1 and 2 of the experiment (i.e. fights were conducted on individuals that had not been fed for 24 h).

### 2.2. Experimental design and procedures

The experiment was designed to explore three objectives, which were to determine: (i) differences in baseline endocrine state between males and hermaphrodites; (ii) whether male–male contests differ in their dynamics from hermaphrodite–hermaphrodite contests; and (iii) whether the endocrine system of males responds differently to contest performance/outcome than that of hermaphrodites. In this study, 72 secondary males (DAN2K, *N* = 52; RHL, *N* = 20) and 72 hermaphrodites (DAN2K, *N* = 52; RHL, *N* = 20) were used. Secondary males naturally transitioned under the common garden conditions described in §2.1, above; they were not experimentally induced to change sex. This experiment was conducted in four sets of 18 trials.

Each set was conducted over two consecutive days. On the day before experimental Day 1, each fish was taken out of the maintenance container and transferred to a transparent plastic bag with a little water, and standard length was measured with calipers. Individuals of the same sex and lineage were divided into similar-sized pairs (difference in standard length ≤1 mm). There was no significant difference in standard length between males and hermaphrodites (hermaphrodite: 27.79 ± 0.02 mm; male: 27.61 ± 0.02 mm; *t*_142 _= −0.62, *p *= 0.535), but there was a significant difference in standard length between RHL and DAN2K (RHL: 28.73 ± 0.03 mm; DAN2K: 27.37 ± 0.02 mm; *t*_142 _= 4.38, *p *< 0.001). On Day 1, pre-contest water-borne hormone samples were collected between 9:30 and 12:10 (see below) and then the two individuals of a pair were marked by cutting the non-vascular thin membrane between the two soft rays in either the upper or lower margins (randomly assigned) of the caudal fin for individual identification. The two fish of a size-matched, sex-matched pair were placed into the two similar-sized compartments of a fighting tank (12 × 8 × 20 cm^3^, filled with room-heated, 25 ppt water and 1–1.5 cm of gravel) and separated by an opaque partition inserted in the middle of the tank for 24 h of acclimation.

On Day 2 between 9:10 and 11:50, the opaque partition was removed to allow the two fish to fight until the contest was resolved with a clear winner and loser. Fights were arranged in a manner where one pair of males and one pair of hermaphrodites of the same lineage fought at the same time. Since there were different sample sizes of DAN2K and RHL lineages, RHL fights were dispersed as equally as possible among DAN2K fights. When contests resolved, or fish did not engage after 20 min, the partition was replaced in the contest tank to separate the fish and end the interaction. All contests were videotaped for behaviour analysis. After the dyadic contest, post-contest water-borne hormone samples were collected. The fish were euthanized with a lethal dose of sodium bicarbonate buffered MS-222 (Finquel^®^), weighed and dissected to obtain gonad mass; gonadosomatic index (GSI) was calculated as gonad mass divided by body mass. Residuals from a general linear regression of body mass against standard length, including all animals, were used as an index of body condition.

### 2.3. Hormone collection, extraction and assay

Procedures for hormone sample collection, extraction and analysis followed Earley & Hsu [40].

Fish were placed in individual glass beakers with 400 ml clean 25 ppt synthetic seawater (one fish/beaker) and allowed to remain in the hormone collection beaker for 1 h exactly. We then used a water-borne hormone collection method to obtain pre-contest and post-contest hormone levels [1,2]. Detailed procedures are described in the electronic supplementary material (S1: Hormone collection, extraction and assay). All hormone data are presented as pg g^−1^ h^−1^. The mean intra-assay coefficient of variation was 4.21% (range: 2.3–6.5%) for testosterone (T); 1.9% (range: 0.9–2.9%) for cortisol (F); 3.4% (range: 2.0–4.9%) for oestradiol (E2); and 3.6% (range: 1.6–6.3%) for 11-ketotestosterone (11-KT). The inter-assay coefficient of variation was 5.2% for T, 4.6% for F, 10.9% for E2 and 11.5% for 11-KT.

### 2.4. Details on staging contests

A fight between hermaphrodites or between males began when the opaque partition separating the contestants was lifted following a 24 h period of acclimation. The contestant that first chased/attacked its opponent for 5 min without retaliation was regarded as the winner; status was confirmed by there being at least two additional aggressive acts delivered by the winner to the loser that went unreciprocated within a 5 min post-contest period. If fish did not engage in a contest, they were transferred to hormone collection beakers after 20 min. The following aggressive behaviours were recorded: (i) latency to first aggressive act: the time at which an individual first initiated an attack; (ii) number of total aggressive behaviours: total attack number before a contest was resolved; (iii) time spent mouth wrestling: both contestants lock mouths and attempt to push their opponent with vigorous caudal fin thrusting, an intense contest behaviour; (iv) escalated contest: individuals engaged in mutual butting, nipping and/or mouth wrestling; and (v) contest duration: elapsed time between when the first contestant initiated display behaviour and the contest resolved.

### 2.5. Data analysis

General linear models were used to examine sex differences in GSI and pre-contest hormone levels. Body condition, age and lineage were included in the model as covariates. General linear models also were used to examine the effects of sex, lineage, and asymmetries between the two contestants in both body condition and pre-contest hormone levels on contest performance (latency to first aggressive act, time spent in mouth wrestling and contest duration). Asymmetries between contestants in the five pre-contest hormones were highly correlated and including them in the models at the same time would result in significant multicollinearity; the effect of each hormone on contest performance was, therefore, tested separately.

General linear mixed models were used to examine the effects of sex, status (winner or loser), lineage and pre-contest hormone levels on total aggressive acts. Body condition was included as a covariate. The interaction terms of sex × status, sex × body condition, sex × pre-contest hormone levels and status × pre-contest hormone levels were also included. We used a within-subjects analysis (status as the within-subjects factor and contest pair as a random effect [49]), because the behaviours of winner and loser were dependent on each other. The correlation of each hormone with aggression was tested separately because pre-contest hormones were highly correlated and including them in the models at the same time would result in significant multicollinearity.

General linear mixed models also were used to examine the effect of sex, status (winner or loser), lineage and contest behaviour on hormone responses. Hormone responses were calculated as the fold change of hormone levels: ln (post-contest hormone level/pre-contest hormone level). Body condition and age were included as covariates. The interaction terms of sex × status, sex × lineage, sex × body condition and status × lineage were included, with the same within-subjects approach as described in the previous paragraph. Because contest behaviours were highly correlated with each other, including them in the models at the same time would result in multicollinearity, therefore the effect of each contest behaviour on endocrine responses was tested separately. Two tailed *t*-tests were used to test the differences of hormone responses in hermaphroditic pairs and male pairs to non-escalated contests and escalated contests.

We were unable to quantify hormone concentrations in a small number of samples (pre-contest T: *N* = 1; post-contest T: *N* = 6; pre-contest F: *N* = 1; post-contest-F: *N* = 4; pre-contest E2: *N* = 2; post-contest E2: *N* = 4; pre-contest 11-KT: *N* = 1; post-contest 11-KT: *N* = 4), and the degrees of freedom for related analyses reflect this. Five contest pairs that did not engage in fights were excluded from behavioural analyses.

Pre- and post-contest hormone levels, time of first aggressive act, number of total aggressive behaviours, time spent in mouth wrestling and contest duration were natural-log (ln) transformed to achieve normality. Relative differences (asymmetry) in hormone levels between two contestants were calculated as: (absolute value of difference between two contestants)/(mean value of two contestants).

We used SAS Enterprise Guide (v. 9.4; SAS Institute Inc., Cary, NC, USA) for the analysis of general linear models (PROC GLM) and general linear mixed models (PROC GLMM); and we used JMP (v. 12; SAS Institute Inc.) for all other statistical analyses in this study.

## 3. Results

### 3.1. Sex differences in GSI and pre-contest hormone levels

**Figure 1.**
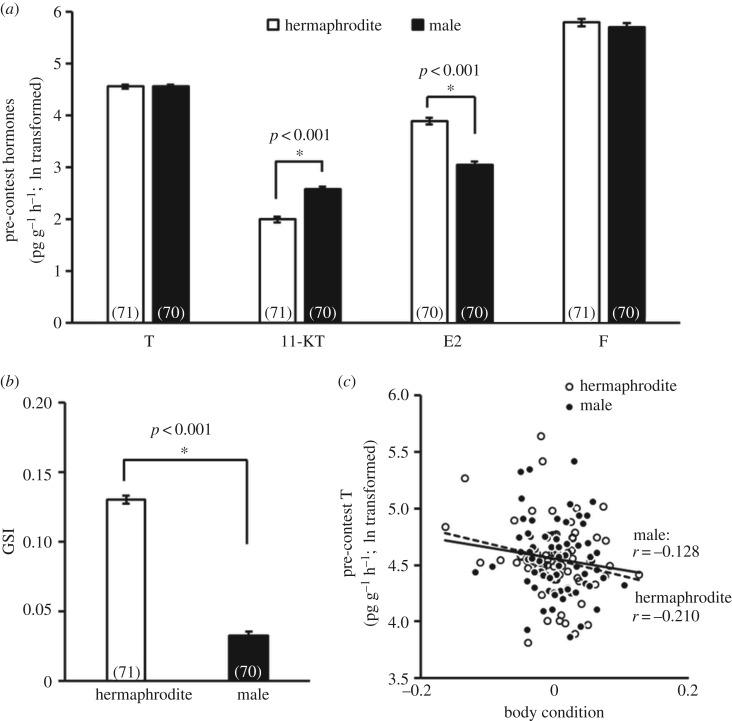
Sex differences in (*a*) pre-contest hormone levels, and (*b*) GSI. (*c*) The relationship between body condition and pre-contest testosterone level. The numbers in brackets represent the sample size of each sex. T, testosterone; 11-KT, 11-ketotestosterone; E2, estradiol; F, cortisol; GSI, gonadosomatic index; white bars and white circles, hermaphrodite; black bars and black circles, male.

Hermaphrodites had significantly higher E2 (*F*_1, 135 _= 33.89, *p *< 0.001, figure 1*a*) and GSI (*F*_1, 136 _= 162.72, *p *< 0.001, figure 1*b*) than males, while males had significantly higher 11-KT (*F*_1, 136 _= 20.40, *p *< 0.001, figure 1*a*) than hermaphrodites. There were no significant sex differences in pre-contest levels of T or F. Individuals that had better body condition also had lower pre-contest T (hermaphrodite: *r* = −0.210, *p* = 0.079; male: *r* = −0.128, *p* = 0.291; overall: *F*_1, 136 _= 4.06, *p *= 0.046, figure 1*c*); body condition was not related to any other hormone.

### 3.2. Sex, lineage and pre-contest hormones as predictors of contest performance

**Figure 2.**
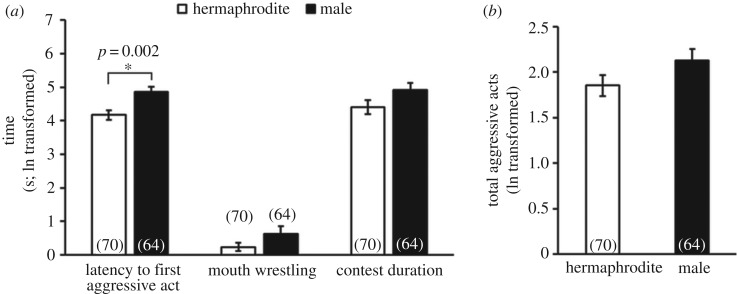
Sex differences in contest behaviour, including (*a*) the latency to first aggressive act, time spent in mouth wrestling, contest duration, and (*b*) total aggressive acts. The numbers in brackets represent the sample size of each sex. White bars, hermaphrodite, black bars, male.

Males took longer than hermaphrodites to initiate the first aggressive act but tended to spend more time mouth wrestling, engage in contests of longer duration (figure 2*a*, electronic supplementary material, table S2) and display more aggressive acts (figure 2*b*, electronic supplementary material, table S3) than hermaphrodites. Contest winners delivered more aggressive acts than contest losers (electronic supplementary material, table S3). Differences in pre-contest E2 between contestants were positively correlated with the latency to first aggressive act (electronic supplementary material, figure S4, table S2); asymmetries between contestants in body condition and in the other pre-contest hormone levels were not related to contest behaviours (electronic supplementary material, table S2). The two isogenic lineages fought in different ways, with RHL engaging in longer fights than DAN2K (electronic supplementary material, figure S5, Table S2). Similar to our previous studies, pre-contest levels of some hormones were highly correlated with individual contest behaviours. Individuals with higher pre-contest T exhibited more aggressive acts during a contest (hermaphrodite: *r* = 0.282, *p* = 0.018; male: *r* = 0.117, *p* = 0.153; overall: *F*_1, 123 _= 6.02, *p *= 0.017, electronic supplementary material, figure S6, table S3). Interactions between status and pre-contest T, F, and 11-KT were significant predictors of contest behaviour (electronic supplementary material, table S3), suggesting that eventual winners and losers showed different relationships between pre-contest hormone levels and total aggressive acts. Pre-contest T and 11-KT of eventual losers, but not eventual winners, were positively correlated with total aggressive acts (electronic supplementary material, figure S7A, S7C, table S3). Pre-contest F of eventual winners, but not eventual losers, was negatively correlated with total aggressive acts (electronic supplementary material, figure S7B and table S3).

### 3.3. Sex, status, lineage and contest behaviour as predictors of hormone responses

**Figure 3.**
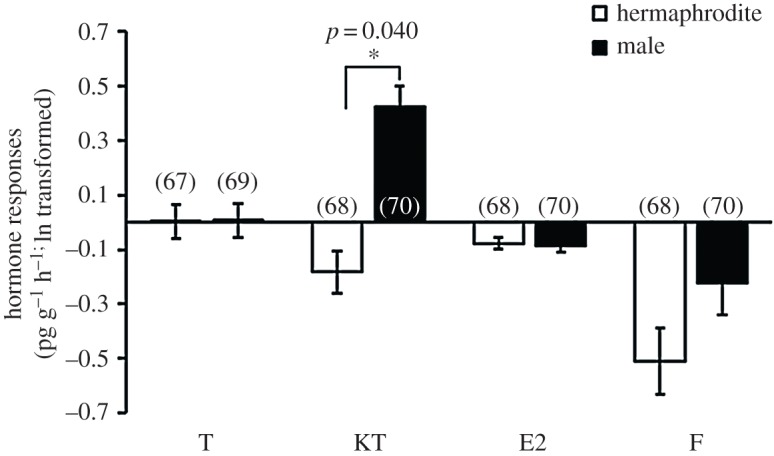
Sex differences in hormonal responses to social challenge. The numbers in brackets represent the sample size of each sex. T, testosterone; 11-KT, 11-ketotestosterone; E2, oestradiol; F, cortisol; white bars, hermaphrodite; black bars, male.

**Figure 4.**
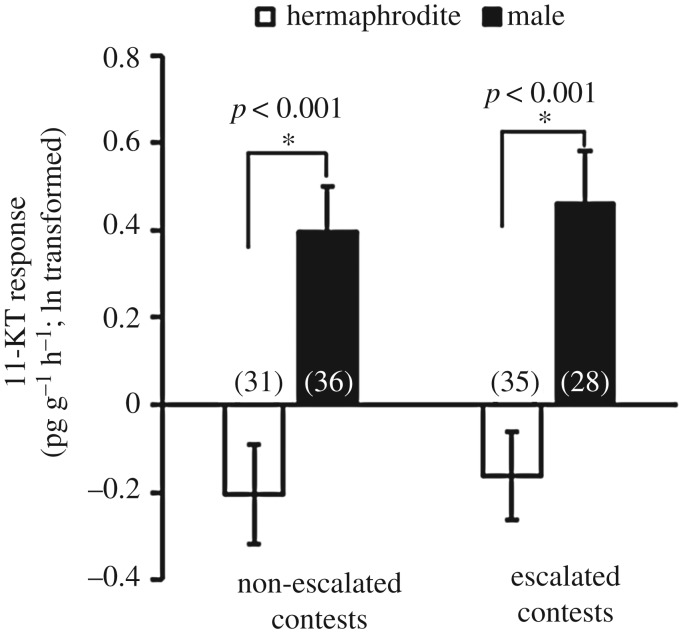
Response of 11-ketotestosterone (11-KT) in hermaphroditic pairs and male pairs to non-escalated contests and escalated contests. The numbers in brackets represent the sample size of each sex. White bars, hermaphrodite; black bars, male.

Sex, lineage and contest behaviour were significant predictors of hormonal responses to fighting, but post-contest hormone levels were not influenced by status (winner versus loser; table 1, electronic supplementary material, figure S8). Males exhibited significantly greater 11-KT responses to contests than hermaphrodites (figure 3, table 1, electronic supplementary material, figure S8), regardless of whether the male had won or lost the contest and despite the fact that they already had higher pre-contest 11-KT than hermaphrodites (figure 1*a*). There were no significant differences between the sexes in T, F or E2 responsiveness to contest experience (figure 3, table 1, electronic supplementary material, figure S8). The sex difference in 11-KT responsiveness was not because males fought more intensely than hermaphrodites; males had a significantly higher 11-KT response than hermaphrodites in both escalated and non-escalated contests (two tailed *t*-test: non-escalated contest: *t*_66 _= 4.01, *p* < 0.001; escalated contest: *t*_62 _= 3.73, *p* < 0.001, figure 4, electronic supplementary material, figure S9). The interaction between sex and lineage was a significant predictor of the 11-KT response (table 1). There was no significant difference between lineages in the 11-KT response of hermaphrodites but male RHL showed a significantly higher response in 11-KT than DAN2K (two tailed *t*-test, hermaphrodite: *t*_65 _= −1.04, *p* = 0.308; male: *t*_63 _= 3.16, *p* = 0.003, electronic supplementary material, figure S10). Body condition was a significant predictor of hormone responses to social challenge. Both hermaphrodites and males with better body condition responded to aggressive contests with a more pronounced increase in all four hormones (table 1, electronic supplementary material, figure S11). The interaction between sex and body condition was not a significant predictor of hormonal responsiveness to social challenge, suggesting that hermaphrodites and males showed the same relationship between body condition and endocrine responsiveness. Both males and hermaphrodites that exhibited more total aggressive acts during contests had lower T responses (hermaphrodite: *r* = −0.236, *p* = 0.059; male: *r* = −0.246, *p* = 0.051; overall: *F*_1, 117_ = 13.73, *p* < 0.001, table 1, electronic supplementary material, figure S12). There were no other significant relationships between contest behaviours and hormonal responses.

**Table 1.** Predictors of hormonal responses to social challenge. Because the contest behaviours were highly inter-correlated, each behaviour was included in a separate model to prevent multicollinearity. T, testosterone; 11-KT, 11-ketotestosterone; E2, oestradiol; F, cortisol; W/L, winner/loser.

		T response		11-KT response		E2 response		F response	
variable	d.f.	*F*	*p*	*F*	*p*	*F*	*p*	*F*	*p*
sex	(1, 117) or (1, 119)	0.19	0.661	4.34	0.040*	0.20	0.659	0.15	0.704
status (W/L)		0.43	0.514	1.35	0.250	0.31	0.581	0.00	0.972
body condition		14.18	<0.001*	5.56	0.022*	10.95	0.002*	7.42	0.008*
lineage		1.01	0.318	1.31	0.256	0.27	0.605	1.49	0.227
age		0.46	0.499	3.91	0.052	0.03	0.853	4.36	0.041*
sex × status		2.77	0.101	1.12	0.295	1.30	0.259	0.40	0.529
sex × lineage		0.76	0.386	5.72	0.019*	0.00	0.958	0.63	0.431
sex × body condition		0.66	0.420	0.61	0.440	0.15	0.698	0.04	0.847
status × lineage		1.29	0.260	0.83	0.365	0.68	0.411	1.14	0.290
latency to first aggressive act	(1, 117) or (1, 119)	1.11	0.296	0.59	0.445	0.46	0.500	0.49	0.487
total aggressive acts	(1, 117) or (1, 119)	13.73	<0.001*	0.03	0.854	0.86	0.358	0.59	0.445
contest duration	(1, 117) or (1, 119)	2.78	0.101	1.41	0.240	0.00	0.954	2.69	0.106

*p < 0.05

## 4. Discussion

### 4.1. Sex differences despite genetic uniformity

We discovered that males and hermaphrodites showed significant differences in baseline hormone levels, contest behaviour and endocrine responses to social challenges despite being genetically identical, suggesting that wholesale changes in gene expression (independent of genomic sequence) accompany sex change. We also revealed that sex differences varied between the isogenic lineages, indicating that genotype also plays an important role in dictating variation in behavioural and hormonal responses to social experience. Given that many species have no sex chromosomes and the developmental fate of the bi-potential gonad is determined by environmental cues experienced during a critical period of larval development [50], it is not surprising that factors other than genotypic differences play important roles in shaping divergent sexual phenotypes. Other species change sex at specific ages/sizes or in response to social stimuli. In a socially stable group of bluebanded gobies (*Lythrypnus dalli*), loss of the dominant male from a social group triggers the dominant female to initiate sex change and produces dramatic behavioural and morphological modifications, including rapid increases in aggression and male courtship behaviour [31,51,52]. Such sex differences might be initiated by environmentally induced alterations to patterns of gene expression in the neuroendocrine system, which might be driven by, or cooperate with, activation or de-activation of sex determination genes to initiate dramatic changes to the sexual phenotype [53–55].

Epigenetic modification is one of most probable mechanisms underlying the emergence of sex differences associated with environmentally-induced sex change. Epigenetic modifications (e.g. DNA methylation or histone modification) mediate expression of genes that contribute to phenotypic diversity without altering the underlying genetic code [56]. Rivulus exist predominantly as self-fertilizing hermaphrodites; primary males arise from exposure to low temperatures (18–20°C) during embryonic development, and secondary males arise from exposure to high temperature (≥28°C) and photoperiodic changes during adulthood [35,57,58]. Ellison *et al*. [59] compared DNA methylation patterns between individuals exposed to different temperatures (18–25°C) during embryonic development and found that low temperature significantly increased the production of primary males and affected DNA methylation patterns. Low temperature also induced significant epigenetic changes in sex determination genes (e.g. aromatase, Sox9a, dmrt1 and foxl2), which are associated with sexual phenotype and may be responsible also for the dramatic sex differences in aggressive behaviour and hormonal responses to social challenges. While we do not know whether primary and secondary males exhibit the same epigenetic profiles, Ellison *et al*.'s [59] data suggest that epigenetic processes might underlie the marked differences in aggressive behaviour and endocrine responses to social challenge between hermaphrodites and secondary males that we revealed in our study.

### 4.2. The effects of sex and status on hormone responses

Recent victories and defeats affect an individual's behaviour and its probability of winning future contests (winner–loser effects) [60]. Numerous studies have shown that winning contests increases an individual's androgen levels or the expression of androgen receptors (genes or proteins); these endocrine responses guide aggressive behaviour, promote winning probabilities in future contests and mediate the winner effect [16,44,45]. However, this endocrine mechanism was not found in hermaphroditic rivulus. Winning and losing experiences changed an individual's future contest behaviour but did not influence androgen or oestrogen hormone levels [41], and altered brain androgen receptor gene expression but only in individuals with low baseline T [45].

We predicted that the endocrine status of males would respond in the expected manner to social experiences but that the hormonal responses of hermaphrodites to contest experiences might be constrained because they must maintain both ovarian and testicular function. Androgen responses to social challenge in hermaphrodites might also be constrained by the limited amount of testicular tissue that they possess (less than 20% of the ovotestis [36]). Our results supported this hypothesis and revealed that contest interactions initiated a significant increase in 11-KT for males but not hermaphrodites. Unlike other studies, this hormone response was not significantly different between winners and losers, and it did not correlate with individual aggressive behaviours performed during the contest. One possible explanation relates to the different experimental procedures used in the aforementioned studies. The experimental designs of other studies often provided focal individuals with territorial advantage [16,44] or let the winners continue to directly (physical contacts) or indirectly (olfactory or visual cues) interact with the losers to reinforce social status [42]. In our study, however, we did not provide focal individuals with territories and completely separated winners and losers after contests were resolved. Our results suggest that contest experiences of any type may drive increases in androgen, particularly 11-KT, levels but that other factors such as post-contest interaction, territorial advantage or reinforcement of dominance status might be responsible for differences in physiological status between winners and losers. Territorial advantage or post-contest interaction may further mediate androgen responses or neuroendocrine status and shape individuals' aggressive behaviour in future contests.

Similar phenomena were discovered in the serotonergic system of other species: both dominant (winner) and subordinate (loser) individuals exhibited significant increases in serotonergic activity during and right after a contest, while the effect persisted significantly longer in losers than in winners as long as dominant individuals continued to interact with subordinate individuals [61–63]. Most studies that investigate physiological responses to social experiences do not discriminate the effect of ‘contest experience’ from ‘post-contest interaction’, which may possibly exaggerate the degree to which acute wins and losses precipitate neuroendocrine change. Our results also suggest that pre-contest T was positively correlated with aggressive behaviour in both males and hermaphrodites. This clear positive correlation between pre-contest T and aggression is similar to our previous studies [40,41], suggesting that T is involved in regulating individuals' aggressive behaviour. However, experience-induced behavioural changes (i.e. winner and loser effects) may not be mediated by androgen levels in rivulus because eventual winners and losers exhibited similar post-contest T and 11-KT levels immediately following the fight.

## 5. Conclusion

Despite the fact that hermaphrodites and males have identical genotypes, this study revealed that (i) *before contests*, hermaphrodites had higher baseline E2 but lower baseline 11-KT than males; (ii) *during contests*, males took longer to initiate contests but fought more aggressively and sustained longer fights than hermaphrodites; and (iii) males had a dramatic post-contest increase in 11-KT but hermaphrodites did not. Genetically identical animals with different sexual phenotypes performed notably different fighting behaviours and exhibited significantly different endocrine responses to social challenge. This raises the question of which epigenetic mechanisms might be responsible for altering gene expression patterns in ways that affect neuroendocrine function and behaviour. Such mechanisms are likely to uniquely define the sexes in rivulus, and be linked in some salient way with the behavioural and physiological differences that we have uncovered in this study.

## Supplementary Material

Supplementary materials

## Supplementary Material

Datasets

## Data accessibility

The datasets supporting this article have been made available as a part of the electronic supplementary material. File name: Li_Male vs. Herm ms_rsos-ESM2_datasets.
