# Supplementary material for: Sexual phenotype drives variation in endocrine responses to social challenge in a quasi-clonal animal
Source: R Soc Open Sci. 2018 Apr 4;5(4):180002. doi: 10.1098/rsos.180002 (PMC5936956; doi:10.1098/rsos.180002)
Supplement: Supplementary materials [file rsos180002supp1.doc]

***Electronic Supplementary Material***

**S1. Hormone collection, extraction and assay**

We used a water-borne hormone collection method to obtain pre-contest and post-contest hormones [1,2]. Fish were placed in individual glass beakers with 400 ml clean 25 ppt synthetic seawater (one fish/beaker) and allowed to remain in the hormone collection beaker for 1h exactly. After 1 hour, fish were removed from the beakers. Hormone was extracted from the water samples by using C18 solid phase extraction columns (Lichrolut C18, 500 mg, 3.0 ml; Merck) fitted to a 24-port vacuum manifold. Columns first were primed with 2×2 ml HPLC grade methanol (MeOH) followed by 2×2 ml distilled water. Water samples were slowly drawn through the collection columns by vacuum using Tygon tubing. When the samples had passed completely through the columns, salts were removed with 2×2 ml washes of distilled water. Columns then were frozen until further processing. Freeze storage of water samples and columns had been determined not to impact steroid concentrations [3]. Columns then were thawed and purged with 2×2 ml washes of distilled water. Hormones were eluted from the columns into 12×75 mm (6 ml) borosilicate vials by 2×2 ml washes with HPLC grade MeOH. The 4 ml of eluted solvent was evaporated at 40 ºC (water bath) with a gentle stream of ultra-pure, nitrogen gas, which was passed over the samples through an evaporating manifold. The resulting hormone residue was resuspended in 800µl of enzyme-immunoassay (EIA) buffer supplied with the kits (see below) and the samples stored at -20°C until assay. Cayman Chemicals Inc. EIA kits were used for all hormones (testosterone [T], cortisol [F], 17β-estradiol [E2] and 11-ketotestosterone [11-KT]), following the manufacturer’s recommended procedures. Plates were read at 405nm on a BioTek microplate reader. Earley and Hsu [4] validated the Cayman Chemicals, Inc. kits for *K. marmoratus* water-extracted hormones by assessing parallelism of a serial dilution curve with the standard curve. Extraction efficiency was assessed by generating a pooled mangrove rivulus water-borne hormone extract with known concentration (i.e., mixing 30µl of the resuspension from each experimental animal), spiking a subsample of the pool with one of four different kit standards, passing the spiked samples over the C18 columns, and processing the samples as described above. The minimum observed recovery (% of expected concentration based on known pool and standard concentrations) was 87.2% for T, 91.3% for F, 90% for E2 and 87.1% for 11-KT.

**Table S2. Sex differences in aggressive behavior during contests.** Asymmetry in body condition, asymmetry pre-contest hormones and lineage were also included in this model as covariates. The interaction terms of “sex **×** asymmetry in body condition” and “sex **×** asymmetry in pre-contest hormones” were also included in this model. Because the asymmetries between contestants in the five pre-contest hormones were highly inter-correlated, each pre-contest hormone was included in a separate model to prevent multicollinearity. (T: testosterone, F: cortisol, E2: estradiol, 11-KT: 11-ketotestosterone, Δ: relative differences/asymmetries between two contestants within each pair ** p < 0.05*)

|  | | **Latency to 1^st^ aggressive act** | | **Mouth**  **wrestling** | | **Contest**  **duration** | |
| --- | --- | --- | --- | --- | --- | --- | --- |
| **Variable** | **df** | ***F*** | ***P*** | ***F*** | ***P*** | ***F*** | ***P*** |
| **Sex** | (1,59)  or  (1,58) | 10.09 | **0.002*** | 1.51 | 0.225 | 2.37 | 0.129 |
| **ΔBody condition** |  | 1.31 | 0.256 | 0.39 | 0.533 | 1.06 | 0.308 |
| **Lineage** |  | 0.33 | 0.567 | 0.03 | 0.870 | 4.25 | **0.044*** |
| **Sex × ΔBody condition** |  | 3.50 | 0.066 | 0.19 | 0.664 | 1.56 | 0.216 |
| **ΔT** | (1,59) | 0.78 | 0.380 | 0.05 | 0.823 | 0.09 | 0.764 |
| **Sex × ΔT** | (1,59) | 0.23 | 0.636 | 1.62 | 0.208 | 0.10 | 0.748 |
| **Δ11-KT** | (1,59) | 0.64 | 0.426 | 0.20 | 0.659 | 2.52 | 0.117 |
| **Sex × Δ11-KT** | (1,59) | 0.05 | 0.816 | 0.48 | 0.492 | 0.44 | 0.509 |
| **ΔE2** | (1,58) | 4.79 | **0.032*** | 0.10 | 0.756 | 0.15 | 0.699 |
| **Sex × ΔE2** | (1,58) | 1.79 | 0.186 | 0.88 | 0.352 | 0.37 | 0.547 |
| **ΔF** | (1,59) | 0.70 | 0.408 | 0.26 | 0.615 | 3.43 | 0.069 |
| **Sex × ΔF** | (1,59) | 0.18 | 0.669 | 0.46 | 0.498 | 1.39 | 0.243 |

**Table S3. Sex and status differences in total aggressive acts during contests.** Body condition, lineage and pre-contest hormones were also included in this model as covariates. The interaction terms of “sex **×** status”, “sex **×** body condition”, “pre-contest hormone × sex” and “pre-contest hormone **×** status” were also included in this model. Because pre-contest hormones were highly inter-correlated, each pre-contest hormone was included in a separate model to prevent multicollinearity. (T: testosterone, F: cortisol, E2: estradiol, 11-KT: 11-ketotestosterone, ** p < 0.05*)

|  | | **Total aggressive acts** | |
| --- | --- | --- | --- |
| **Variable** | **df** | ***F*** | ***P*** |
| **Sex** | (1,122)  or  (1,123) | 1.85 | 0.179 |
| **Status (W/L)** |  | 51.01 | **<.001*** |
| **Body condition** |  | 0.00 | 0.990 |
| **Lineage** |  | 0.26 | 0.613 |
| **Sex × Status (W/L)** |  | 0.00 | 0.982 |
| **Sex × Body condition** |  | 0.13 | 0.721 |
| **Pre-T** | (1,123) | 6.02 | **0.017*** |
| **Pre-T × Sex** | (1,123) | 0.00 | 0.994 |
| **Pre-T × Status (W/L)** | (1,123) | 5.99 | **0.017*** |
| **Pre-11-KT** | (1,123) | 1.97 | 0.166 |
| **Pre-11-KT × Sex** | (1,123) | 0.03 | 0.863 |
| **Pre-11-KT × Status (W/L)** | (1,123) | 5.74 | **0.020*** |
| **Pre-E2** | (1,122) | 0.91 | 0.344 |
| **Pre-E2 × Sex** | (1,122) | 0.10 | 0.751 |
| **Pre-E2 × Status (W/L)** | (1,122) | 2.22 | 0.141 |
| **Pre-F** | (1,123) | 1.75 | 0.190 |
| **Pre-F × Sex** | (1,123) | 3.25 | 0.076 |
| **Pre-F × Status (W/L)** | (1,123) | 4.89 | **0.031*** |


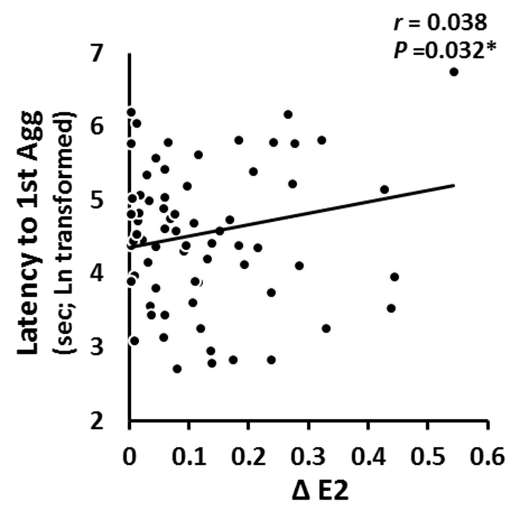
**Figure S4.** The relationship between latency to first aggressive act and the difference in pre-contest estradiol between two contestants. (Δ: relative differences/asymmetries between two contestants within each pair; E2: estradiol)


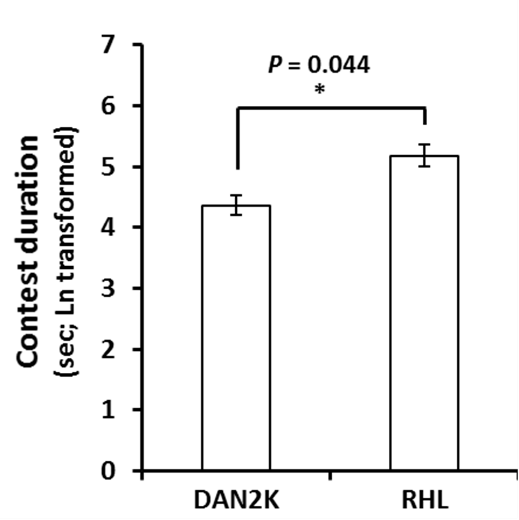


**Figure S5.** Lineage difference in contest duration.

**
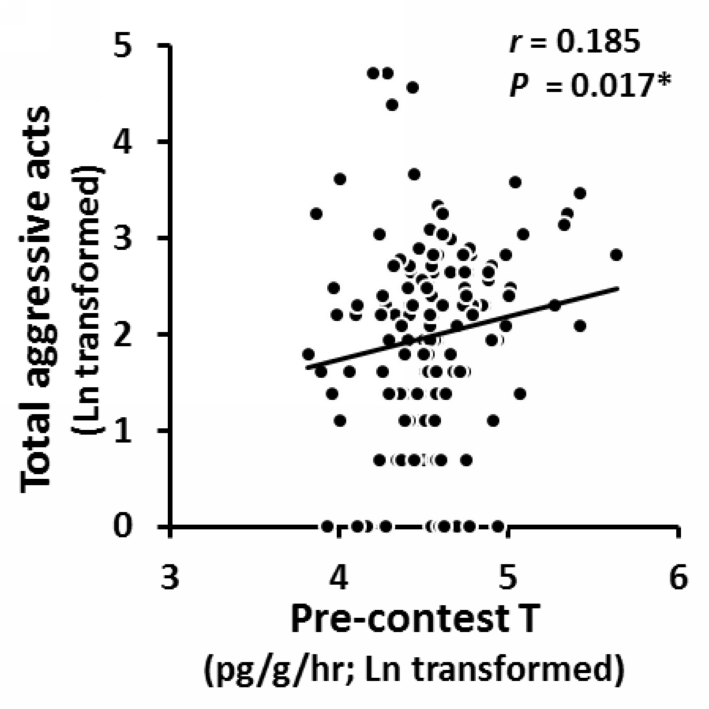
Figure S6.** Relationship between pre-contest testosterone levels and total aggressive acts. (T:
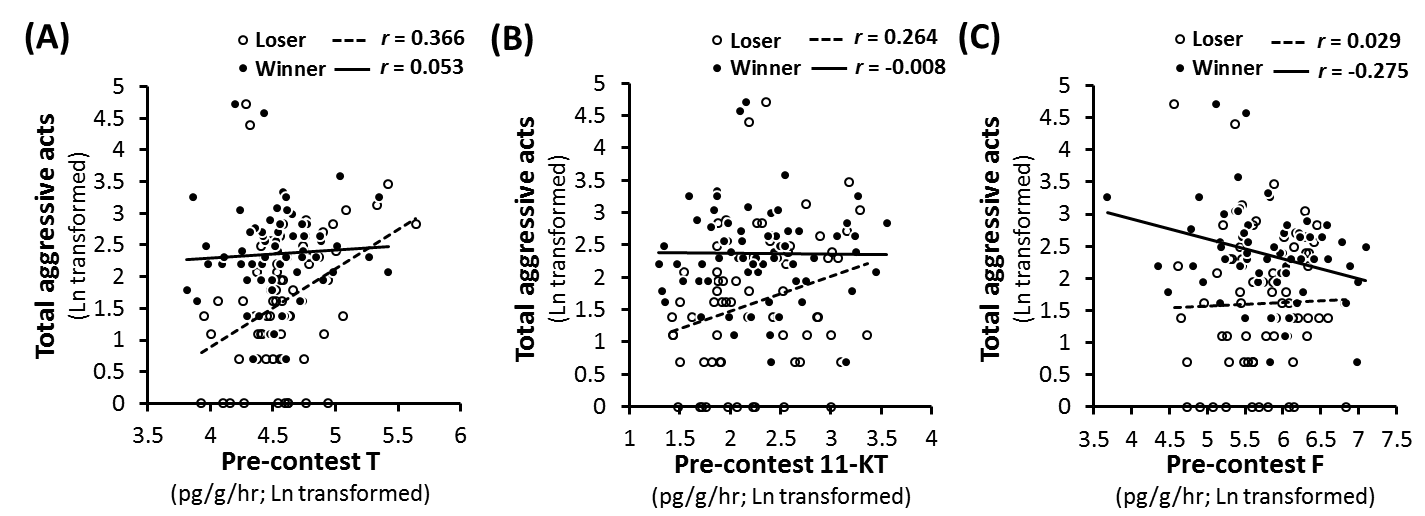
testosterone)

**Figure S7.** Winner and loser show different relationships (A) between total aggressive acts and pre-contest testosterone; (B) between total aggressive acts and pre-contest cortisol; (C) between total aggressive acts and pre-contest 11-ketotestosterone. (T: testosterone, F: cortisol, 11-KT: 11-ketotestosterone)

**Figure S8**
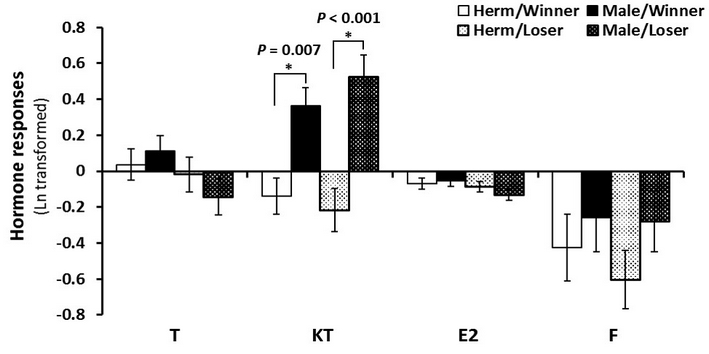
**.** Sex and status differences in hormonal responses to social challenge. (White bars: hermaphroditic winner, black bars: male winner, white branch bars: hermaphroditic loser, black branch bars: male loser)


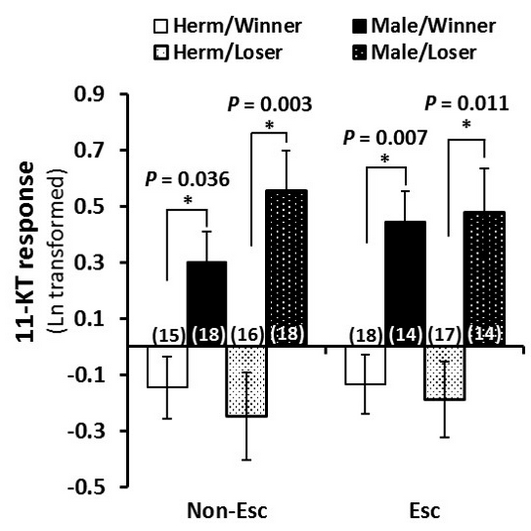


**Figure S9.** Response of 11-KT in hermaphroditic pairs and male pairs to non-escalated contests (Non-esc) and escalated contests (Esc). (White bars: hermaphroditic winner, black bars: male winner, white branch bars: hermaphroditic loser, black branch bars: male loser)


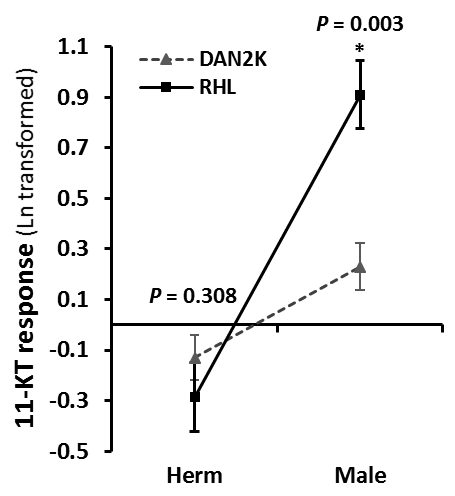

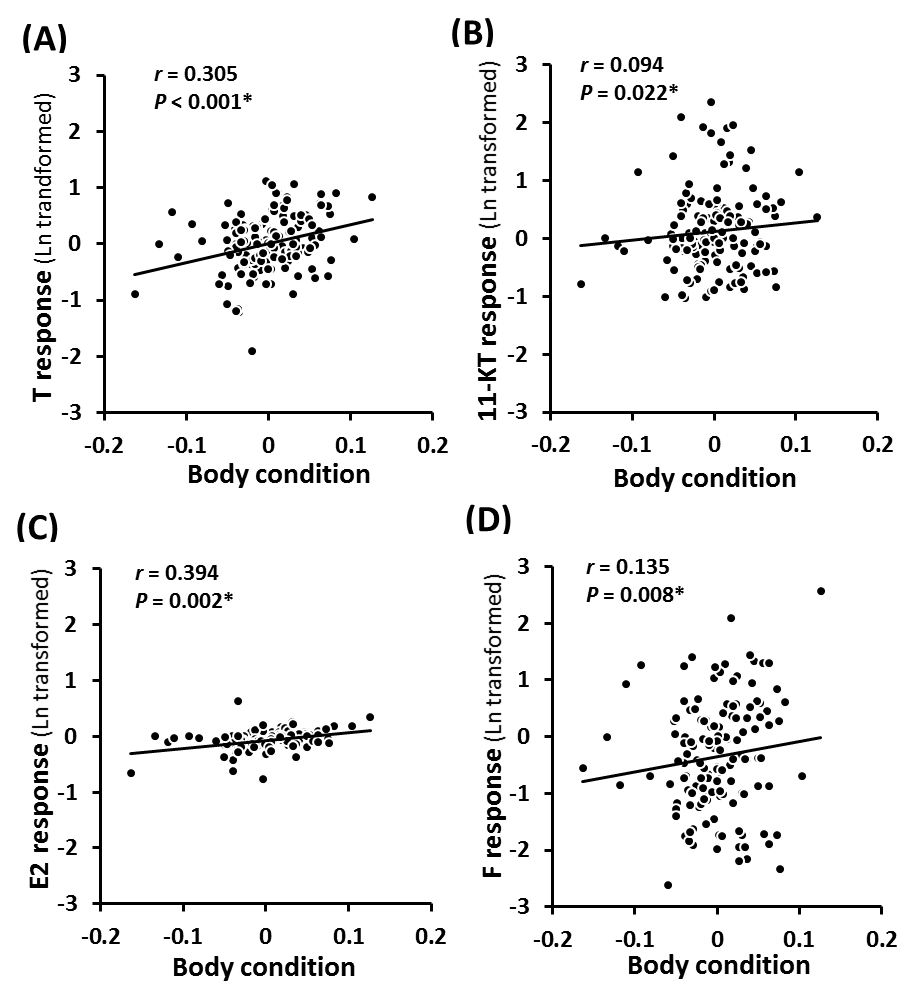
**Figure S10.** Differences between lineages in 11-ketotestosterone responsiveness to social challenge for both hermaphroditic contest pairs and male contest pairs. (11-KT: 11-ketotestosterone)

**Figure S11.** Relationships between body condition and hormonal responses to social challenge. (T: testosterone, F: cortisol, E2: estradiol, 11-KT: 11-ketotestosterone)


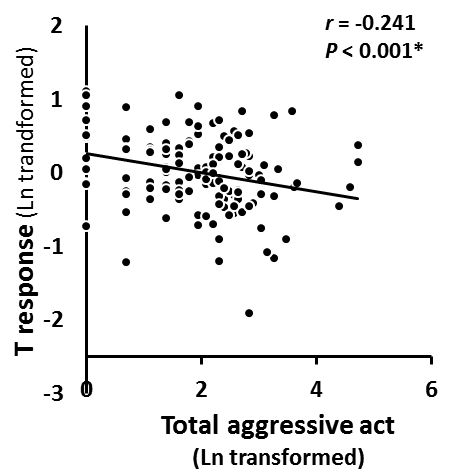
**Figure S12.** The relationship between the total aggressive acts performed during the contest and responsiveness of testosterone to social challenge. (T: testosterone)

***References***

1. Scott AP, Hirschenhauser K, Bender N, Oliveira RF, Earley RL, Sebire M, Ellis T, Pavlidis M, Hubbard P, Huertas M, Canario AVM. 2008 Non-invasive measurement of steroids in fish holding water: important considerations when applying the procedure to behaviour studies. *Behaviour* **145**, 1307-1328. (doi:10.1163/156853908785765854)
2. Ellis T, Sanders MB, Scott AP. 2013 Non-invasive monitoring of steroids in fishes. *Vet. Med. Austria* **100**, 255-269.
3. Ellis T, James JD, Stewart C, Scott AP. 2004 A non-invasive stress assay based upon measurement of free cortisol released into the water by rainbow trout. *J. Fish Biol.* **65**, 1233-1252. (doi:10.1111/j.0022-1112.2004.00499.x)
4. Earley RL, Hsu Y. 2008 Reciprocity between endocrine state and contest behavior in the killifish, *Kryptolebias marmoratus*. *Horm. Behav.* **53**, 442-451. (doi:10.1016/j.yhbeh.2007.11.017)
